# Effects of Cold Pressor Stress on the Human Startle Response

**DOI:** 10.1371/journal.pone.0049866

**Published:** 2012-11-15

**Authors:** Christian E. Deuter, Linn K. Kuehl, Terry D. Blumenthal, André Schulz, Melly S. Oitzl, Hartmut Schachinger

**Affiliations:** 1 Department of Clinical Psychophysiology, Institute of Psychobiology, University of Trier, Trier, Germany; 2 Department of Psychiatry and Psychotherapy, Charité University Medicine, Berlin, Germany; 3 Department of Psychology, Wake Forest University, Winston-Salem, North Carolina, United States of America; 4 Research Unit INSIDE, Division of Clinical and Health Psychology, University of Luxembourg, Luxembourg, Luxembourg; 5 Division of Medical Pharmacology, Leiden/Amsterdam Center for Drug Research and Leiden University Medical Center, University of Leiden, Leiden, The Netherlands; University of Sydney, Australia

## Abstract

Both emotion and attention are known to influence the startle response. Stress influences emotion and attention, but the impact of stress on the human startle response remains unclear. We used an established physiological stressor, the Cold Pressor Test (CPT), to induce stress in a non-clinical human sample (24 student participants) in a within-subjects design. Autonomic (heart rate and skin conductance) and somatic (eye blink) responses to acoustic startle probes were measured during a pre-stress baseline, during a three minutes stress intervention, and during the subsequent recovery period. Startle skin conductance and heart rate responses were facilitated during stress. Compared to baseline, startle eye blink responses were not affected during the intervention but were diminished afterwards. These data describe a new and unique startle response pattern during stress: facilitation of autonomic stress responses but no such facilitation of somatic startle eye blink responses. The absence of an effect of stress on startle eye blink responsiveness may illustrate the importance of guaranteeing uninterrupted visual input during periods of stress.

## Introduction

Stress is regarded to be an adaptive reaction to an adverse stimulus or situation. The stress response is a multi-level, complex shift in the organism’s physiological and psychological functioning [1]. The physiological stress response allocates bodily resources to facilitate quick, evasive actions at the expense of more long term, regenerative functions. Acute stress involves an endocrinal response [2] and activation of the sympathetic nervous system [3], and influences somatic motor behaviour and psychological adjustments.

In contrast to the low-level, biological adaptations, that meet the change in energy demands, stress effects on basic psychological processes, and interaction with attention and emotion, are less well understood. Some studies found an attentional bias for aversive, threatening stimuli under stress exposure [4], [5], while others found no [6], [7] or even opposing effects, with less attention for negative stimuli after stress manipulation [8], [9]. However, differences of independent and dependent variables used in the above cited studies complicate the search for answers. The experimental paradigm of startle eye blink modification may provide a biology-based measure of emotional and attentional effects that might clarify these questions.

The startle response is a fast defensive mechanism that protects the organism against potential injury. Elicited by abrupt and intense stimuli in various sensory modalities, the startle response protects the organism against imminent physical harm in a natural setting, e.g. due to a predator or a blow [10]. Somatic muscle contractions and activation of the autonomic nervous system (ANS) represent the two major components of the response. Sudden contractions of facial and flexor skeletal muscles induce a defensive posture and protect essential parts of the body. Acceleration of heart rate and increased skin conductance responses (SCR) indicate an activation of the ANS and prepare the organism for action, e.g. fight or flight [11].

The emotional context in which startle is elicited may modulate the response magnitude in one of two directions. Positive emotional states attenuate and negative states enhance the startle response. Such affective startle modulation has been explained in terms of motivational priming: aversive emotional stimuli prime the defensive motivational system and thereby facilitate defensive reflexes, whereas appetitive emotional stimuli inhibit defensive reflexes [12]. Experimental paradigms have employed a diverse array of emotional stimuli with positive or negative hedonic valence, such as pictures [13], films [14], music [15], odours [16],or anxiety-inducing darkness [17]. Also, placebo effects of neutral stimuli [18] and the imagination [19] or anticipation [20] of emotional content modulate the startle response.

However, although it is typical that psychological ratings indicate a negatively valenced emotional stress response, e.g. increased ratings of adversity, irritability, anxiety, and loss of control, the effect of stress on startle responsiveness remains unclear. A prototypical laboratory stressor is the well defined Cold Pressor Test (CPT) [21], [22]. De Peuter et al. [23] found potentiated startle responses during a one minute CPT. While this result is consistent with motivational priming, other studies found opposing effects. Tavernor et al. [24] used a 90 s CPT and found lower startle magnitudes in the CPT condition. However, the ice water hand immersion during the CPT was rather brief in both studies, as compared to earlier studies in which immersion lasted up to 6 min. [25], [26]. With such a time schedule, only three startle noise presentations, a very limited number in human startle research, were delivered during the CPT in the Tavernor et al. study. Considering that CPT stress effects may need some time to develop, e.g. the first 30 s of ice water hand immersion are often well tolerated, and strongest blood pressure increases appear during the second minute [27], [28], [29], [30], it may be speculated that not all startle probes were delivered during a genuine stress experience of the participants, thus making the time course of effects incomparable to De Peuter’s study. In the current study we aimed to investigate the effects of a longer lasting (3 min) CPT version on the human startle response, with special focus on the different startle response components, e.g. somatic motor vs. autonomic responses.

Somatic motor reactions occur faster than changes of the autonomic nervous system (ANS). Motor startle reactions have been shown in various human muscle systems such as facial [31], cervical, or limb muscles [32]. Indicators of the autonomic startle response are cardiovascular [33], [34] and SCR changes [35]. Interestingly, the magnitudes of these response components do not always show common variation. Such response separation is shown, for example, by the fact that startle eye blink responses exhibit differences in habituation from SCR [36], [37] and cardiovascular startle responses [38]. So far, it is unclear whether stress affects startle ANS and somatic motor responses in a similar way, or whether it may induce a response separation.

Acute stress effects may carry over into the recovery period. Since we wanted to compare pre, during, and post stress effects, we decided to use equally long periods (3 min) with an equal number of startle probes before (pre), during, and after (post) the CPT intervention. We measured autonomic startle responses in heart rate and SCR. The eye blink response was measured by recording the electromyographic (EMG) response of the orbicularis oculi muscle, and we also measured the actual eyelid movement via video recordings. This method allowed us to study the kinematics of the startle eye lid movement, which is essential to identify the consequences of the startle eye blink response for the continuation of the visual signal input flow.

## Methods

### Participants

Twenty-four undergraduate students of the University of Trier participated in this study. Participants were interviewed for actual and past medical and/or psychiatric health problems. Resting blood pressure (BP) (Dinamap System, Critikon, US) was assessed. Exclusion criteria were acute or persistent medical and psychiatric diseases, current medication except the occasional use of pain killers (paracetamol, aspirin, or NSAR), actual or past hearing problems (e.g. tinnitus), a history of fainting, and BP greater than 140/90 mmHg or systolic BP lower then 110 mmHg.

Four participants were excluded from further analysis because of complete loss of startle eye blink responsiveness during the initial habituation phase (“nonresponder”). The final sample included 20 participants (11 f/9 m, mean age = 24.29 y, SD = 2.53 y).

Participants gave a written informed consent and were financially compensated with 15€ for participation. Experimental procedures were approved by the ethical committee of the medical association of Rhineland-Palatinate.

### Experimental Design

Each participant was subjected to both conditions in two separate blocks: stress (Cold Pressor Test, CPT) and control intervention (hand immersion in warm water), with a resting break of 45 minutes in between the two blocks. The sequence of conditions was counterbalanced between subjects.

**Figure 1:**
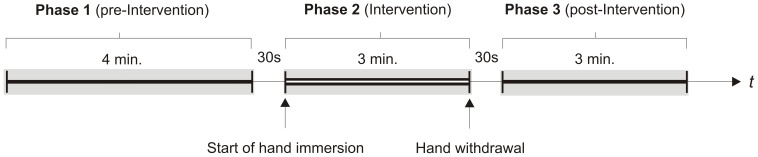
Experimental protocol.

Each block was divided into three phases: pre-intervention (phase 1; 4 min), intervention (phase 2; 3 min), and post-intervention (phase 3; 3 min). In phase 1, 12 startle probes were presented, in each of the other phases 8 startle probes were presented (see figure 1).

### Stress Intervention

The CPT was used to elicit a physiological stress response. The participant’s right hand was placed in a bucket with crushed ice and water up to the wrist. Water temperature ranged between 0° and 4°C. Participants were instructed to leave the hand in the water for a period of three minutes.

The control condition was structured identically to the stress condition, with the only difference being that the water temperature was near body level (35°C).

### Startle Stimulation

Startle stimuli were acoustic white noise probes (105 dB, 50 ms duration, instantaneous rise time, binaural stimulation) presented via audiometric headphones (Holmco PD-81, Holmberg GmbH & Co. KG, Germany). Startle probes were presented with a variable inter-stimulus-interval of 10 to 16 s. The first four startle probes during phase 1 served as habituation trials and were not included in further analysis. Initial startle responses are usually exaggerated in size. After a few trials, habituation follows a more gradual course. Therefore it is common to exclude initial trials from further analysis (“habituation trials”) [42]. We could verify this by statistically comparing the habituation trials to the baseline (‘phase 1′) trials,

### Procedure

Experiments were performed in the afternoon between 2 and 6 p.m. After being checked for exclusion criteria and signing their consent form, participants were familiarized with the laboratory setting. They were seated in front of an eye tracker (SMI iView-X HiSpeed 500), mounted on a height-adjustable table, with a 19′′ - TFT-monitor (1280×800 resolution). The distance between eye tracker and monitor was 35 cm. The participant was instructed to adjust to a comfortable sitting position. Electrodes were attached and the participant placed the head in a stable position, with forehead and chin resting on the eye tracking device throughout the experiment. The eye tracker was calibrated to control for the participant’s gaze position and headphones were adjusted.

The experiment started with an instruction appearing on the screen, informing the participant that the experiment would start and to sit quietly, keep their eyes in the direction of a fixation cross that would appear in the middle of the screen, and neither move nor speak. The experiment started with the grey fixation cross appearing on a black screen. This cross remained on the screen throughout the experiment.

Subsequent to phase 1, the experimenter took the participant’s hand and placed it in the water bucket. After three minutes, the experimenter removed the hand, dried it with a towel and placed it on the table again. The participants gaze remained on the screen throughout the experimental intervention. Phase 3 started 30 sec. after the end of the water immersion.

### Data Acquisition and Analysis

#### Electromyography

Electrodes for EMG recording of the m. orbicularis oculi were attached below the participant’s right eye at an inter-electrode distance of 1.5 cm. The EMG-signal was recorded on hard disk with a BIOPAC MP 150 system and an EMG 100C amplifier via Tyco Healthcare H124SG electrodes at 16 bit resolution and 1 kHz sampling rate. Hardware band-pass filter settings were 10 to 500 Hz, followed by a 28 Hz software high-pass filter [39]. The raw signal was rectified and integrated online with a time constant of 10 ms [40].

The EMG-startle responses were analyzed offline with a C++based, semi-automated program. Startle response was defined as difference between peak and baseline signal. The integrated algorithm identified peak in a time interval between 20–150 ms after stimulus onset. Baseline was assessed 50 ms prior to stimulus onset [41]. Each response was manually confirmed and corrected for non-responses and artefacts. Non-responses (cases with no discernable response) were set to zero and included in the analysis (1.09% of all responses). Cases with electrical and physiological artefacts (such as voluntary or spontaneous eye blinks coinciding with the startle stimulus, or trials with excessive background noise or multiple peaks) were excluded from analysis (2.83% of all responses). Responses were averaged across participants for each condition. Zero response data were included in the averaging procedure, with startle response magnitude as the final output measure [42].

#### Image eye blink recording

The motion of the left eyelid was assessed with an image based approach. We used a different eye for the two measures, to assess the eye blink kinematics without the possible interference of invasive electrode placement. With binaural stimulation, we deemed it safe to assume that laterality effects can be ignored [42].

The start of image recordings was synchronized with the onset of the acoustic startle probe. Each recording sequence lasted 600 ms. The images were recorded at a frequency of 500 Hz, thereby generating 300 images per trial. All images were of 256 bit depth greyscale resolution; image size was 224×160 px. Pictures were assessed with the internal eye camera of the eye tracker and a customized version of the iView recording software (SMI iView 2.5, SensoMotoric Instruments GmbH, Teltow, Germany). The images were automatically saved to the hard disk of the eye tracking computer.

Image analysis was conducted manually by measuring the distance between upper and lower eyelid on the mid pupil position in the picture at startle probe onset and in the picture with the maximum eye closure. Lid distance was measured in pixels with a digital ruler (Pixel Ruler 4.0, Mioplanet, Rimouski, Canada). Maximal eye closure was expressed as the percentage of eye lid closure at the point of maximal closure in relation to baseline lid distance at the beginning of the trial. Responses were averaged across participants for each phase.

In addition to being the baseline for blink quantification, initial lid distance (aperture) is reported as a measure of muscle tone of the upper eyelid.

#### Cardiovascular data

Electrodes for ECG-measurement (ECG Tyco Healthcare H34SG Ag/AgCl electrodes, diameter: 45 mm) were placed according to a standard lead II configuration. The signal was acquired with the BIOPAC MP 150 and a ECG100 Amplifier. The signal was high-pass filtered (0.5 Hz, hardware filter) and stored to disk (1 kHz). Beat-to-beat heart rate data were calculated by a semi-automatic QRS detection in WinCPRS (Absolute Aliens Oy, Turku, Finland). Responses were averaged across participants for each condition.

Heart rate was calculated in beats per minute (bpm). We analysed the mean heart rate values for each condition as well as the startle heart rate response, defined as the change between the time period 4 to 6 s post-startle and the −2 to 0 s pre-startle baseline before startle stimulus presentation.

#### Skin Conductance Responses (SCR)

Skin conductance was measured with BIOPAC MP 150 and a GSR100 Amplifier. Electrodes were the same as for ECG. Electrodes were placed on the palm of the left hand, the signal filtered with a 10 Hz low pass filter.

For SCR analysis we employed the same program that we used for EMG analysis. The response was defined as the peak in a time period of 4–6 s post startle stimulus. Baseline was measured as the mean in the period 2 s before the startle probe. All individual SCRs were log-transformed, and then normalized [Z(log (1+SCR)] per participant. Averaging was done per phase, condition, and participant.

#### Subjective ratings

After the experiment was finished, participants were asked to rate the degree of unpleasantness of the experimental manipulation on a Likert scale ranging from 0 (‘not at all unpleasant’) to 8 (‘very unpleasant’).

### Statistical Analysis

EMG response, eye lid response, skin conductance response, heart rate response, and mean heart rate were analysed in a repeated measures 3×2 ANOVA, with the factors ‘time’ (phase 1: pre-intervention, phase 2: intervention, phase 3: post-intervention) and ‘treatment’ (CPT vs. control) for each dependent variable.

The interaction term is reported, as well as ‘a priori’ defined contrasts reflecting the intervention effects. Contrasts were constructed between phase 1 and phase 2 for CPT vs. the control condition. This contrast reflects the cold pressor stress effect. In a similar way, the post-stressor effects were analysed: contrasts between phase 1 and phase 3 for CPT vs. the control condition. P-values for factors with more than two conditions are reported after Greenhouse–Geisser correction.

Subjective ratings of unpleasantness were analysed for an effect of the level of stressor with a Student’s *t*-test for paired samples.

The critical alpha-level was set to.05 in all analyses.

## Results

### Subjective Ratings

The CPT was rated as significantly more unpleasant than the control condition (t_18_ = 15.48; p<.001, see Table 1).

**Table 1:** Effects of treatment on physiological and subjective parameters: Mean (SD).

	CPT			Control				
	Phase 1	Phase 2	Phase 3	Phase 1	Phase 2			Phase 3
Eye Lid Distance - blink (nadir during blink)	46.81 (29.9)	38.99 (32.2)	39.14 (27.49)	47.24 (27.07)	36.88 (29.26)			41.3 (29.84)
Eye Lid Distance - baseline (initial aperture)	50.7 (6.92)	53.09 (6.02)	50.55 (7.2)	52.09 (5.32)	49.17 (6.04)			48.57 (6.76)
Heart Rate	68.36 (12.02)	75.95 (14.77)	67.59 (11.87)	68.76 (10.61)	69.27 (11.16)			69.16 (10.32)
Probability of Complete Blink	21 (34)	19 (33)	14 (25)	18 (33)	15 (31)			17 (36)
Subjective Ratings for Unpleasantness		6.37 (1.30)				0.89 (0.87)		

*Eye Lid Distance - blink*: distance covered as percent of baseline distance; *Eye Lid Distance - baseline*: pixel; *Heart Rate:* bpm;

*Probability of Complete Blink*: percent; *Subjective Ratings:* 8-digit Likert scale.

### Mean Heart Rate

During the CPT intervention, we found an increase in mean heart rate, which was not present in the control condition. The ANOVA revealed a significant overall interaction between “treatment” (CPT, control intervention) × “time” (pre, during, post intervention) on mean HR (F _2,38_ = 17.58; p<.001; η^2^ = .48), Contrasting HR from phases 1 (pre) and 2 (during) over the intervention blocks, revealed increasing HR during the CPT (F _1,19_ = 13.63; p<.01; η^2^ = .42). There were no statistically significant effects with regard to pre-post intervention differences (see Table 1).

### Initial Eyelid Distance

Compared to the control condition, we found an increased initial eyelid distance during the intervention in the CPT condition. The contrast between phases 1 (pre) and 2 (during) over the intervention blocks, revealed increased initial eyelid distance during the CPT (F _1,19_ = 5.18; p<.05; η^2^ = .21) (see Table 1).

### Startle EMG Response

Startle EMG magnitude was significantly reduced during intervention (F _1,19_ = 8.85; p<.001; η^2^ = .89). The ANOVA revealed a significant overall interaction between “treatment” (CPT, control intervention) × “time” (pre, during, post intervention) on startle EMG responses (F_2,38_ = 4.54; p<.05; η^2^ = .11). There were no statistically significant differences nor interactions considering the kind of treatment (CPT vs. control) during the intervention (phase 2).

**Figure 2:**
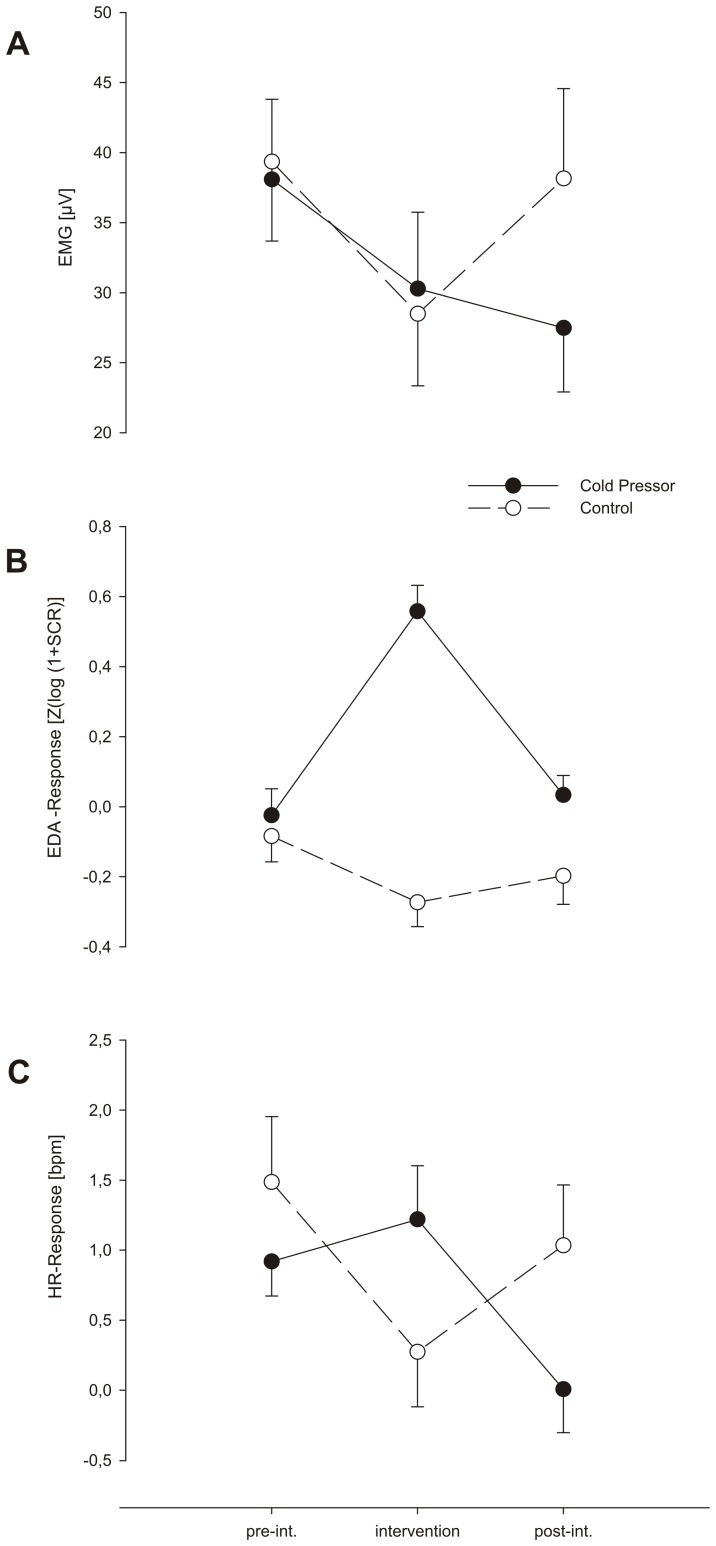
Measures of the startle response: eye blink (EMG)-, skin conductance and heart rate responses.

However, after the intervention, we found lower response magnitudes in the CPT condition then in the control condition. This is expressed in the contrast between phases 1 (pre) and 3 (post) over the intervention blocks (F_1,19_ = 7.13; p<.05; η^2^ = .19) (see figure 2A ).

### Startle Eyelid Response

No significant main effects, nor an interaction between “treatment” (CPT, control intervention) × “time” (pre, during, post intervention) was found for the startle eyelid responses (see Table 1).

### Startle Skin Conductance Response

We found a significant overall interaction between ”treatment” × “time” (F_2,38_ = 4.81; p<.05; η^2^ = .20). Startle SC responses increased during the intervention in the CPT condition, while no such effect was found for the control condition, as expressed in the contrast from phases 1 (pre) and 2 (during) over the intervention blocks (F_1,19_ = 6.19; p<.05; η^2^ = .25). There were no statistically significant effects with regard to pre-post intervention differences (see figure 2B ).

### Startle Heart Rate Response

We found a significant overall interaction between “treatment” × “time” on startle HR responses (F _2,38_ = 4.98; p<.05; η^2^ = .21). Startle Heart Rate Responses increased during the intervention in the CPT condition, while no such effect was found for the control condition, as expressed in the contrast between phases 1 (pre) and 2 (during) over the intervention blocks (F _1,19_ = 5.41; p<.05; η^2^ = .22). There were no statistically significant effects with regard to pre-post intervention differences (see figure 2 C).

## Discussion

In this study, we investigated the influence of cold pressor stress on somatic motor and autonomic components of the human startle response. We identified a unique stress modulation pattern of startle response components, not seen before during experimental manipulation of emotion or attention. The pattern consists of selectively enhanced autonomic startle reactivity, as would be expected during aversive emotional states, but not somatic motor eye blink responsiveness. As such, stress supports individual adjustments to startling danger sources by boosting ANS effects (e.g. on energy supply), but avoiding excessive eye blinks which compromise the continuity of visual input.

The CPT intervention of the current study proved to be successful; our participants rated the CPT intervention as significantly more unpleasant than the control intervention, thereby confirming the subjective aversive component of the stress intervention. Mean heart rate was significantly elevated during the CPT intervention and returned to baseline afterwards, indicative of a sustained autonomic activation during the CPT. This is in line with previous findings (e.g. [43], and can be attributed to increased cardiac sympathetic activity [29].

Startle-evoked autonomic responses were significantly affected by the stress intervention. Startle skin conductance responses were increased during the CPT. Such responses are indicative of higher sympathetic activation and could be expected based on previous studies (e.g. [35]). During the stress intervention a similar pattern of enhanced heart rate startle response was found. However, heart rate responses were lower in the recovery period after the stress intervention. The lowered responsiveness after the CPT could be interpreted as a counter regulatory mechanism: heart rate might decrease as a result of increased vagal outflow, which is mediated by enhanced baroreflex sensitivity in response to sustained blood pressure increases [44].

The CPT had a different effect on startle-evoked somatic motor responses. The CPT manipulation did not reveal a significant difference for EMG-measured orbicularis oculi muscle activity during the intervention. Compared to the pre-intervention baseline, startle magnitude was significantly reduced in both conditions,.Subsequent to the intervention, post-stress startle magnitude was significantly lower compared to baseline in the CPT condition. This post-stress effect is comparable to the above described startle heart rate response. However, the EMG response pattern of m. orbicularis oculi activity did not translate into actual kinematic eyelid response. For this measure, no differences were found.

The EMG response pattern demands further explanation. In the presence of differential autonomic responses during the CPT intervention, we found no difference in the eye blink response during the CPT. Motivational priming would predict a potentiation of eye blink startle in an unpleasant state. However, it may be the case that different aspects of the intervention had a differential influence on the startle response. Attentional processes could possibly counteract affective modulation. Considering the more intense stimulation in the cold pressor condition, as compared to the control condition, we would expect that more attentional resources are directed to the stressor. This would imply that more attention is channelled to thermoceptive and nociceptive input, making attention less available for auditory processing. Directing attention towards the startle eliciting modality can increase startle, whereas directing it to a different modality can reduce startle magnitude [45]. Attentional and emotional factors interact and may work in opposite directions, making the net effect on startle responsiveness difficult to predict [46]. If they are equal in size and point in opposite directions, no observable net effect would appear. This may have been the case for the startle eye blink EMG response. The affect-related increase and the decrease due to attentional focussing would cancel each other out, ultimately provoking the same response magnitudes as in the control condition.

We found lower EMG eye blink responses in the recovery phase, after the intervention was terminated. With the cessation of the experimental manipulation after phase 2, attentional capture can be ruled out as a possible explanation while affective factors still have an impact. In fact, this pattern is in line with the motivational opponent-process theory [47]: during the recovery from an emotional stimulation, valence is predicted to reverse. The cold pressor test has a strong negative valence due to its subjective painfulness, which is known to increase during the time course [48]. Relief itself is highly pleasant and rewarding [49], [50]. This offers an explanation for the attenuated EMG startle response after relief from the unpleasant stressor. The result also corresponds to a study by Franklin et al. [51], that employed the CPT (120 s) as a proxy for non-suicidal self-injury. Startle response magnitude was taken as a measure of cognitive–affective regulation (with pre-pulse inhibition reflecting the cognitive component). That study focused specifically on the pain component of the CPT, actually treating the CPT as a way to gain relief from a previous social-stress intervention. Franklin et al. also found reduced startle after the CPT. However, startle responses were measured only after, but not during, the CPT intervention in that study.

Furthermore, the kinematic analysis of eye lid movements did not reveal a stress effect. This is interesting, since video-measured eye lid movements shows a high correlation with the EMG [52] and startle evoked eye lid closure was susceptible to other experimental manipulations, such as affective startle modulation [53] or prepulse inhibition [54]. We also controlled for the probability of complete blinks (when the eyeball is fully covered by the lid), since these cases can diminish the correlation between lid movement and EMG. However, the probability was low and did not vary between phases or conditions. One possible reason for the absence of an effect in this measure is the impact of muscles other than the orbicularis oculi.The upper eyelid movement is accomplished by the interplay of two skeletal muscles, the orbicularis oculi muscle and the levator palpebrae muscle, as well as the smooth Müller’s muscle, that runs from the musculus levator palpebrae to the upper margin of the tarsal plate. While a blink is basically accomplished by rapid activation of the orbicularis oculi muscle, the other two play a crucial role in lid elevation and upper eyelid tone [55], [56]. Since Müller’s muscle is sympathetically innervated [57], stress (e.g. as in the CPT) could possibly influence the upper eyelid’s muscle tonus and thereby also the movement during the blink. Indeed, we could demonstrate that the initial lid distance (measured as the aperture at the beginning of each trial) was increased during the CPT intervention, which was not the case in the control condition. This would support the hypothesis that stressful situations require a continuation of visual input to process potential threats.

The decrease in magnitude after the intervention could only be found in the EMG eye blink measure, not for the startle ANS responses. We are not able to explain the mechanism underlying such a response discrepancy. However, depending on the situational context, separate response components are weighted differently. Acute stress induces a large scale shift in attentional processing, with increased alertness and activation in defense-related processing structures [58]. Even though the startle related lid closure is adaptive by protecting the sensitive eye in the face of danger, it has detrimental effects as well - for a brief, but potentially crucial moment, visual input is interrupted. Guaranteeing continuous visual input during periods of stress offers some adaptive potential, since it may allow for more rapid and directed defence and escape behaviour. This result would also be in line with a recent study that found a dissociation between autonomic and electrophysiological responses to a CPT, indicating regulatory processes that preserve sensory perception [59]. Therefore, the blink magnitude might reflect a compromise between the need for protecting this vital organ on the one hand and not hindering appropriate action on the other hand.

Some limitations of this study need to be addressed. The validity of the results might be restricted to CPT-induced stress. The CPT distinguishes itself from other interventions in that it is a representative autonomic stressor with a specific activation of the sympathetic nervous system [41], [44], [60], [61]. An additional reason to choose this intervention was the feasibility of video based blink recording without the participant being visually distracted. Still, how these results generalize to other stressful interventions remains open to further research. The degree to which the post-stress effects are mediated by humoral factors could not be addressed in this study. For that purpose, extending this paradigm over a longer post-stress time period would be of interest.

In conclusion, we have demonstrated that cold pressor stress has an effect on the acoustic startle response in humans. However, different components (somatic eye blinks, ANS responses) of the response are differentially affected. The resulting unique pattern of responses would allow for the benefits of ANS adjustments, but still guarantee the continuous input of visual signals.
